# Developing health service delivery in a poor and marginalised community in North West Pakistan

**DOI:** 10.12669/pjms.343.15168

**Published:** 2018

**Authors:** Heather Only, Helen Bingley, Nicola Lowe, Rashid Mehdi, Zia Ul Haq, Mukhtiar Zaman

**Affiliations:** 1 Heather Ohly, University of Central Lancashire, Preston, UK; 2 Helen Bingley, Abaseen Foundation, Lancaster, UK. University of Central Lancashire, Preston, UK; 3 Nicola Lowe, University of Central Lancashire, Preston, UK; 4 Rashid Mehdi, Khyber Medical University, Peshawar, Pakistan; 5 Zia Ul Haq, Institute of Public Health, Khyber Medical University, Peshawar, Pakistan; 6 Mukhtiar Zaman, Lady Reading Hospital, Peshawar, Pakistan

**Keywords:** Maternal, Child, Health, Service, Community, Capacity

## Abstract

**Objectives:**

To improve maternal health and reduce child mortality through developing health service delivery in a poor and marginalised community in North West Pakistan.

**Methods:**

This was a multifaceted intervention to extend and strengthen the range and quality of services provided at an existing health centre, in a rural community in Peshawar District, Khyber Pakhtunkhwa Province. The intervention was developed with community involvement and had four main components: service development, staff capacity development, community engagement and the introduction of a micro-credit scheme. The evaluation assessed the efficiency and effectiveness of project implementation, including a survey of maternal and child health indicators.

**Results:**

Between 2014 and 2017, a range of new health services were developed at the health centre. Local volunteers were trained to promote health awareness in the community and refer pregnant women to the health centre. The survey indicated health improvements, such as increased vaccination rates for women and children, and a dramatic reduction in unskilled deliveries.

**Conclusions:**

Community engagement was essential to achieve much needed maternal and child health improvements in this poor and marginalised community. Sustainability was achieved by training local volunteers as community health workers.

## INTRODUCTION

Pakistan was recently ranked 122^nd^ out of 157 countries for progress towards the United Nations’ Sustainable Development Goals.^1^ Goal 3 (Good Health and Well-being) includes targets to reduce the global maternal mortality ratio to less than 70 per 100,000 live births by 2030, and all countries to reduce under-5 mortality to at least as low as 25 per 1,000 live births by 2030.^2^ Pakistan has much work to do, with maternal mortality at 178 per 100,000 live births and under-5 mortality at 81.1 per 1,000 live births.^1^ These figures have not yet reached the targets set by the Millennium Development Goals (2000-15). In 2010, a report highlighted several areas of concern in Pakistan: the proportion of births attended by skilled personnel (41%), the proportion of women attending antenatal sessions (58%) and the use of contraceptives (31%).^3^

In response to these concerns, the UK Department For International Development (DFID) funded a three-year project (2014-17) to improve maternal health and reduce child mortality through developing health service delivery in a poor and marginalised community in North West Pakistan. This paper presents evaluation findings and lessons learnt from this project, which we hope will be useful for other researchers and practitioners working to improve health service delivery in similar settings.

### Local Setting

The project was implemented in a rural brick kiln community in Peshawar District, Khyber Pakhtunkhwa Province. This community includes Afghan refugees, internally displaced people and the host population (approximately 100,000 people in total) who mostly work as labourers in the brick kilns. Many households subsist on less than one US dollar per day. The adult female literacy rate is around 3%. Girls typically marry in their early teens and have multiple pregnancies with short birth spacing.

Until 2010, this community had limited access to affordable local health services. An unpublished health survey conducted by the Abaseen Foundation^1^ in 2009 (200 households) found high levels of acute malnutrition (26.6%) and chronic malnutrition (43.1%), poor uptake of antenatal care (14.3%), low levels of infant and child immunisation (20%) and financial problems faced at delivery (89%). In 2010, the Abaseen Foundation^2^ was contracted to manage a local health centre built by HOPE’87 (Hundreds of Original Projects for Employment, Austria) with donations from ANT Hiroshima (Japan) on land donated by the community. In the second and third year of operation, DFID provided funding for essential health services, including antenatal and postnatal care.

### Intervention Approach

The Abaseen Foundation (AF) is a collaboration between two organisations, a non-governmental organisation based in Peshawar, Pakistan (AFPK), supported by its sister Charitable Incorporated Organisation (AFUK) based in Lancashire, UK.

**Table-I:** Summary of survey findings.

Indicator	Baseline	Follow-up
Satisfaction with the range and quality of services provided at the health centre	36%	77%
Women used some form of family planning	18%	27%
Women followed a birth preparedness plan	32%	65%
Pregnant women received Tetanus vaccination	20%	80%
Women had unskilled deliveries	50%	5%
Women experienced problems during delivery	68%	57%
Children under 5 immunised against BCG	60%	77%
Households kept animals in the home	84%	42%

This DFID-funded project (2014-17) aimed to extend and strengthen the range and quality of services provided at the health centre, and in the community, with the long-term goal to improve maternal health and reduce under-5 mortality.

Project design was informed by a series of consultation activities, including a survey (n=1043 households), rapid needs assessment (interviews with 8 key informants) and meetings with community leaders (as part of an unpublished ethnographic study) to elicit the most important health needs of vulnerable groups within the community.

The intervention was multifaceted, with four main components:

- Service development including reproductive health, immunisations, gynaecological, safe delivery and nutrition services;
- Staff capacity development including professional staff and volunteers;
- Community engagement including the formation of village health committees (locally known as *Jirga*) to promote awareness and assist with health campaigns and referrals to the health centre;
- Introduction of a micro-credit scheme to provide financial support to pregnant women (equivalent to 12 GBP) to cover the cost of ultrasound, transport expenses, medicine and delivery charges.

### Project Evaluation

The evaluation assessed the efficiency and effectiveness of project implementation. Data were collected on the number of beneficiaries and services delivered. A survey was conducted by the Institute of Public Health, Khyber Medical University at baseline (2014) and follow-up (2016). It included 60 households purposively recruited from 1000 households living within a 1.5 km radius of the health centre. The survey assessed a range of maternal and child health indicators. However, it did not assess longer-term effects on maternal health and under-5 mortality. Focus groups were conducted with 12 beneficiaries (6 female and 6 male) at the end of the intervention period.

## RESULTS

During the three-year intervention period (2014-17), a total of 79,358 beneficiaries were reached, including 11,159 women of reproductive age (15 to 49 years) and at least 11,712 children under 5 (incomplete data). A total of 98 staff were trained: 22 professional staff (1 doctor, 2 nurses and 19 support staff) and 76 volunteers recruited from the local community (36 outreach workers and 40 traditional birth attendants).

The role of the volunteer staff was to promote health awareness in the community (with support from *Jirga*). Outreach workers made 13,386 household visits to promote awareness of the services available at the health centre. They also conducted 158 health and hygiene sessions in schools (96 male schools and 62 female schools). Traditional birth attendants made 1850 household visits to deliver health education to women, including reproductive health and personal hygiene. They referred 513 pregnant women to the health centre. A nutritionist conducted 639 nutrition education sessions with women and children. A total of 1238 women were registered onto the micro-credit scheme and issued with vouchers.

At the health centre, 7315 children under 5 were screened for malnutrition, of which 1056 were enrolled and treated. Likewise, 5404 pregnant and lactating women were screened for malnutrition, of which 243 were enrolled and treated. The health indicators that improved between 2014 and 2016 are shown in Table-I.

However, some health indicators did not show improvements, including antenatal visits, breastfeeding rates and use of nutritional supplements during pregnancy. The nutritional supplements were not provided free of charge (this was not budgeted for) and most families could not afford them.

During the focus groups, the 12 beneficiaries expressed overall satisfaction and appreciation for the services provided at the health centre and in the community. However, two women said the doctor was absent when they attended the health centre and they had to return several times. They reported that the micro-credit scheme made a big difference to the affordability of health care in such a poor community. However, in some cases, the payments did not cover the whole cost of skilled deliveries.

## DISCUSSION

This project made considerable progress towards improving maternal health and reducing child mortality in the brick kiln community. The combination of clinical services, community services and financial support was embraced by the local community. Trust was developed through engagement with village health committees and heads of households, which enabled pregnant women to attend the health centre.

Capacity building was one of the main achievements of this project. Training 76 local women as outreach workers and traditional birth attendants was a notable achievement in such a conservative, patriarchal community. Most of these volunteers have continued in the same roles since the intervention ended in 2017. This supports previous evidence on community health workers as a sustainable approach to improving population health in low-income countries, including the Lady Health Worker Program in Pakistan.^4^,^5^ Training modules from the LHW Program were used to train the volunteers in this community.

Further efforts are needed to increase awareness of the importance of family planning, antenatal visits, exclusive breastfeeding up to six months, and immunisations for children under 5. Staff and beneficiaries suggested that future developments at the health centre could include 24-hour services, an ambulance, a female doctor, and provision of malaria treatment. Staff also highlighted some challenges experienced during the intervention. The power supply to the health centre was unreliable (long and frequent interruptions) and this was resolved by installing solar panels on the roof. The rural location of the brick kiln community is not easily accessible for professional staff, most of whom live in Peshawar. Furthermore, the roads to the intervention area are in poor condition, which makes access problematic for staff and patients.

## Summary of lessons learnt

- Community engagement was essential to achieve much-needed maternal and child health improvements in this poor and marginalized community.
- The micro-credit scheme helped families to afford better health care for pregnant women and skilled deliveries.
- Sustainability was achieved by training local volunteers as community health workers.

## References

[1] Sachs J, Schmidt-Traub G, Kroll C, Durand-Delacre D, Teksoz K (2017). SDG Index and Dashboards Report 2017.

[2] United Nations Development Programme. Sustainable Development Goals.

[3] (2010). Government of Pakistan (2010) Pakistan Millennium Development Goals Report 2010.

[4] Bhutta Z A, Lassi Z S, Pariyo G, Huicho L (2010). Global Experience of Community Health Workers for Delivery of Health Related Millennium Development Goals:A Systematic Review, Country Case Studies, and Recommendations for Integration into National Health Systems.

[5] Perry HB, Zulliger R, Rogers MM (2014). Community Health Workers in Low-, Middle-, and High-Income Countries:An Overview of Their History, Recent Evolution, and Current Effectiveness. Annu Rev Public Health.

